# Benchmark solution for the stability of plane Couette flow with net throughflow

**DOI:** 10.1038/s41598-021-90552-5

**Published:** 2021-05-25

**Authors:** B. M. Shankar, I. S. Shivakumara

**Affiliations:** 1 Department of Mathematics, PES University, Bangalore, 560 085 India; 2 Department of Mathematics, Bangalore University, Bangalore, 560 056 India

**Keywords:** Engineering, Mathematics and computing

## Abstract

This paper investigates the stability of an incompressible viscous fluid flow between relatively moving horizontal parallel plates in the presence of a uniform vertical throughflow. A linear stability analysis has been performed by employing the method of normal modes and the resulting stability equation is solved numerically using the Chebyshev collocation method. Contrary to the stability of plane Couette flow (PCF) to small disturbances for all values of the Reynolds number in the absence of vertical throughflow, it is found that PCF becomes unstable owing to the change in the sign of growth rate depending on the magnitude of throughflow. The critical Reynolds number triggering the instability is computed for different values of throughflow dependent Reynolds number and it is shown that throughflow instills both stabilizing and destabilizing effect on the base flow. It is seen that the direction of throughflow has no influence on the stability of fluid flow. A comparative study between plane Poiseuille flow and PCF has also been carried out and the similarities and differences are highlighted.

## Introduction

The stability of plane Poiseuille flow (PPF) where both the plates are at rest, plane Couette flow (PCF) where both upper and lower plates are moving in opposite directions and plane Couette-Poiseuille flow (PCPF) where the lower plate is at rest and the upper plate is in motion in its own plane have been the subject of many investigations from the past many decades. Much of the work on these problems has been concerned with the question of whether or not these flows are stable with respect to arbitrary infinitesimal disturbances and for all Reynolds numbers, however large, is still not definitely settled. The Orr-Sommerfeld equation for the flow associated with both PPF (Orszag^1^) and PCPF (Potter^2^) yield instability.

Early attempts to solve the Orr-Sommerfeld equation for the flow associated with PCF led to the prediction of stability for all values of Reynolds numbers, in apparent disagreement with the experiment, although the prediction is correct for infinitesimal disturbances. The chronological developments on PCF are well documented in the book by Drazin and Reid^3^. It was believed that PCF is stable for infinitesimal disturbances, as first suggested by Rayleigh^4^. Numerical studies of the linear problem by Grohne^5^, Deardorff^6^, Davey^7^ and Gallagher^8^ provided valuable insight into the general modal structure of the problem but, again, no instability was found. However, the first general proof of stability seems to be due to Romanov^9^, who proved that the normal modes of the linear problem are damped for all values of Reynolds and wave numbers. The above works offered a fertile ground for further developments; examples include the transverse magnetic field (Kakutani^10^, Takashima^11^), the inclusions of non-Newtonian effects (Gorodtsov and Leonov^12^, Lee and Finlayson^13^, Renardy and Renardy^14^, Wilson et al.^15^, Kupferman^16^, Eldabe et al.^17^) and porous medium domain (Shankar et al.^18^). These studies revealed the possibility of fluid flow becoming unstable.

One of the important and well-known methods for the control of instability of flow in a channel is to inject and withdraw the fluid with the same rate through the walls, known as throughflow or crossflow. The effect of throughflow is, in general, quite complex and, it distorts the basic velocity distribution and also adds additional convective terms to the Orr-Sommerfeld equation of stability. The effect of throughflow on the stability of PPF and PCPF has been studied by many authors due to its wide range of applications in the biomedical industry such as filtration and flow in artificial kidneys, in cooling-heating applications and in the boundary layer control areas. The fundamental model on this problem was first developed by Hains^19^ and showed that crossflow instills stabilizing effect on both PPF and PCPF. In particular, the critical Reynolds number was found to be identical irrespective of the direction of throughflow due to symmetry in the basic state of PPF, but not true in the case of PCPF. In addition, PPF offers a more stabilizing effect compared to PCPF in the presence of throughflow. A similar study was considered by Sheppard^20^ on PPF in the presence of throughflow and showed that throughflow stabilizes the basic channel flow. Later, Fransson and Alfredsson^21^ dealt with the same problem with a slightly different approach, wherein they considered the stability of a shear flow allowing it to be both a function of the mean velocity distribution and the Reynolds number separately. In particular, they used the maximal channel velocity as their velocity scale, and thus separated the effects of base velocity magnitude from those of the base velocity distribution. Interestingly, they demonstrated that the effect of throughflow could be either stabilizing or destabilizing depending on the values of governing parameters. Guha and Frigaard^22^ presented a linear stability of the PCPF in the presence of throughflow. They observed that the destabilization of the basic flow as the throughflow velocity is increased and then stabilization at large values of throughflow velocity and also, the energy analysis was executed to decipher the mechanism of instability. The stability of PPF with net throughflow and an adverse temperature gradient was studied by Bajaj^23^ and showed that the effect of throughflow is to stabilize or destabilize the base flow depending upon its magnitude as well as direction and the Prandtl number. Recently, Shankar and Shivakumara^24^ investigated the effect of a uniform vertical throughflow on the stability of porous-Poiseuille flow and found that the throughflow dependent Reynolds number instills both stabilizing and destabilizing effects on the base flow.

To the best of our knowledge, the effect of a uniform vertical throughflow on the hydrodynamic stability of PCF against small disturbances has not been investigated so far, and the objective of this investigation is to furnish the missing information. It is well known that the PCF is always stable for all the values of Reynolds number so far as small disturbances are concerned with no throughflow. At first glance, it is natural to assume that the PCF in the presence of throughflow will also be stable against small disturbances for all the values of Reynolds number. However, the present investigation has revealed that this conjecture is not always true because the basic velocity profile is distorted by the effect of throughflow and permits the unstable mode of disturbance. The stability of fluid flow is analyzed by solving the stability eigenvalue problem numerically using the Chebyshev collocation method.

## Formulation of the problem

**Figure 1:**
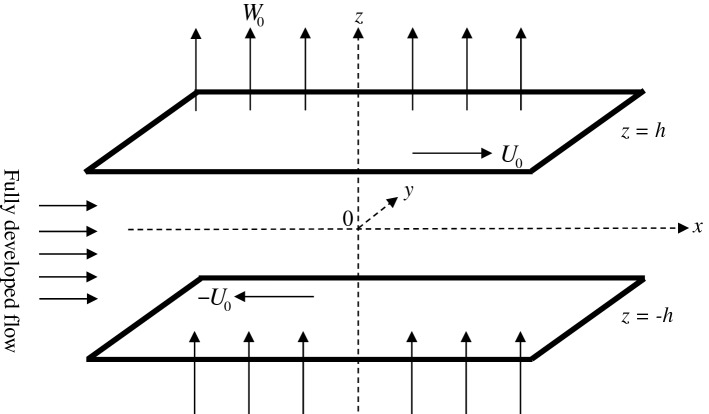
Schematic illustration of the physical problem.

We are considering an incompressible viscous fluid bounded by two horizontal rigid, however permeable, plates which are moving in the opposite directions with a uniform speed $U_{0}$ and in the presence of a uniform vertical throughflow of strength $W_{0}$. The flow is considered to be only due to the motion of the plates as well as throughflow and not due to the applied pressure gradient. The sketch in Fig. 1 is three-dimensional wherein the vertical coordinate $z$ spans the limited interval $[ - h,\,\,h]$, while the ($x$, $y$)-coordinates are unbounded and varies over all possible real values. A dimensionless form of the governing equations, given by the local mass balance and the local momentum balance, can be formulated as (Drazin and Reid^3^)

1 $$ \nabla \cdot \vec{q} = 0, $$

2 $$ \frac{{\partial \vec{q}}}{\partial t} + \left( {\vec{q} \cdot \nabla } \right)\vec{q} = - \nabla p + \frac{1}{R}\nabla^{2} \vec{q}, $$

where the coordinates $(x,y,z)$ are non-dimensionalized in the usual way by the half-width of the channel $h$, the fluid velocity $\vec{q} = (\,u,v,w\,)$ by $U_{0}$, the pressure $p$ by $\rho U_{0}^{2}$ and the time $t$ by $h/U_{0}$, where $U_{0}$ is the velocity at the center line of the channel and $\rho$ is the fluid density. Here, $R = U_{0} \,h/\nu$ is the Reynolds number, while $\nu$ is the kinematic viscosity.

The boundaries are rigid but permeable and are moving with a uniform speed in the opposite directions. Hence, the formulation of the problem sketched in Fig. 1 leads to the dimensionless boundary conditions

3 $$ u = \pm 1,\,\,v = 0,\,\,w = {\frac{R_T}{R}} {\text{at}}\,\,\, z = \pm 1. $$

## Base flow

The base flow is fully developed, unidirectional, steady and laminar with a constant vertical throughflow of magnitude $W_{0}$, i.e. $\vec{q} = \vec{q}_{b} = [u_{b} (z),0,R_{T} /R]$ where, $R_{T} = W_{0} h/\nu$ is the throughflow dependent Reynolds number with the convention that the direction of throughflow is upward (downward) if $R_{T}$ is positive (negative), the suffix *b* serves to denote the basic flow and the pressure is constant throughout the fluid. With these assumptions, the *x*-component of the momentum equation is

4a $$ \frac{{d^{2} u_{b} }}{{dz^{2} }} - R_{T} \frac{{du_{b} }}{dz} = 0. $$

The boundary conditions are

4b $$ u_{b} = \pm 1\;\;{\text{at}}\;\;z = \pm 1. $$

The solution of Eq. (4a) subject to Eq. (4b) is

5 $$ u_{b} (z) = \frac{{1 + e^{{2R_{T} }} - 2e^{{(1 + z)R_{T} }} }}{{1 - e^{{2R_{T} }} }}. $$

**Figure 2:**
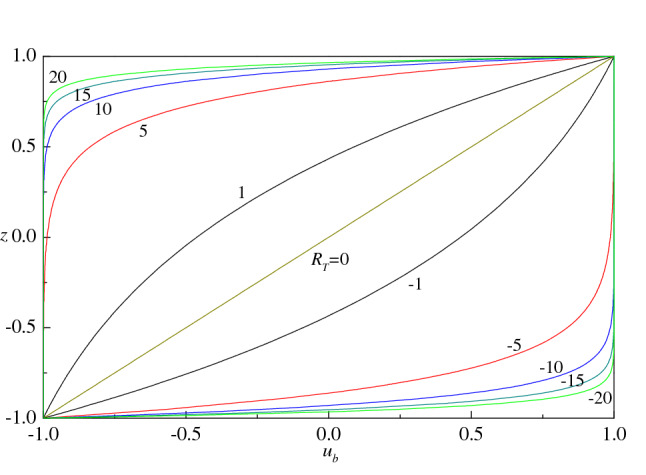
Basic velocity profiles for different values of $R_{T}$.

The plots of $u_{b} (z)$ as a function of $z$ for different values of $R_{T}$ are displayed in Fig. 2. In the absence of throughflow, the basic velocity reduces to the well-known linear profile as expected i.e. $\lim_{{R_{T} \to 0}} u_{b} = z$ (Drazin and Reid^3^). The presence of vertical throughflow, however, changes the basic flow drastically, which has important implications on the stability of fluid flow. It is seen from the figure that an increase in the value of $\left| {R_{T} } \right|$ is to compress the basic velocity profiles toward the boundaries to which the flow is directed. In particular, a very thin boundary layer structure develops close to $z = {{ \pm 1} \mathord{\left/ {\vphantom {{ \pm 1} 2}} \right. \kern-\nulldelimiterspace} 2}$ when $\left| {R_{T} } \right|$ is very large. Figure 2 shows an evident symmetry between positive and negative values of $R_{T}$, when the absolute value of this parameter is fixed. In fact, there is symmetry of solution (5) yielding an invariance under the transformation

6 $$ \vec{q}_{b} \to - \vec{q}_{b} ,\,\,z \to - z,\,\,R_{T} \to - R_{T} . $$

## Linear stability analysis

To discuss the linear stability of fluid flow, the field variables are split into a basic state and an infinitesimal disturbance as

7 $$ \vec{q} = u_{b} \hat{i} + \left( {{{R_{T} } \mathord{\left/ {\vphantom {{R_{T} } R}} \right. \kern-\nulldelimiterspace} R}} \right)\hat{k} + \vec{q}^{\prime } ,p = p_{b} + p^{\prime } , $$

where the primes denote the perturbed quantities. Substituting Eq. (7) into Eqs. (1) and (2) and neglecting the nonlinear terms, we obtain

8 $$ \nabla \cdot \vec{q}^{\prime } = 0, $$

9 $$ \frac{{\partial \vec{q}^{\prime } }}{\partial t} + \left( {\vec{q}^{\prime } \cdot \nabla } \right)\left[ {u_{b} \hat{i} + \left( {{{R_{T} } \mathord{\left/ {\vphantom {{R_{T} } R}} \right. \kern-\nulldelimiterspace} R}} \right)\hat{k}} \right] + \left\{ {\left[ {u_{b} \hat{i} + \left( {{{R_{T} } \mathord{\left/ {\vphantom {{R_{T} } R}} \right. \kern-\nulldelimiterspace} R}} \right)\hat{k}} \right] \cdot \nabla } \right\}\vec{q}^{\prime } = - \nabla p^{\prime } + \frac{1}{R}\nabla^{2} \vec{q}^{\prime } . $$

The stability problem involves the growth of arbitrary infinitesimal perturbations, but it has been generally assumed that such perturbations can be resolved into independent wave-like components. Each component is supposed to satisfy the linearized equations of motion and boundary conditions separately. So we consider a typical wave component with

10 $$ \left( {\vec{q}^{\prime } ,p^{\prime } } \right) = \left( {\vec{q},\,p} \right)(z)e^{{i\left( {\alpha x + \beta y - \alpha ct} \right)}} , $$

where $\alpha$ and $\beta$ are the wave numbers in the $x$ and $y$-directions, respectively and $c = c_{r} + ic_{i}$ is the complex wave speed. It is to be understood that the real part has to be taken to get physical quantities, this being permissible for the linear problem i.e. the flow is stable, neutrally stable or unstable for $c_{i} < 0,\,\,c_{i} = 0$ or $c_{i} > 0$, respectively. Equation (10) serves to separate the variables and reduce the linearized governing Eqs. (8) and (9) from partial to ordinary differential equations and the pressure is eliminated from the momentum equation so that we are led to the following stability equation

11 $$ \left[ {D^{2} - \left( {\alpha^{2} + \beta^{2} } \right)} \right]^{2} w - R_{T} D\left[ {D^{2} - \left( {\alpha^{2} + \beta^{2} } \right)} \right]w = i\alpha R\left\{ {\left( {u_{b} - c} \right)\left[ {D^{2} - \left( {\alpha^{2} + \beta^{2} } \right)} \right]w - D^{2} u_{b} w} \right\}, $$

where $D = d/dz$. The term with coefficient $R_{T}$ has arisen due to the vertical transport of perturbation velocity. The appropriate boundary conditions are

12 $$ w = Dw = 0\,\,\,{\text{at}}\,\,z = \pm 1. $$

Equations (11) and (12) forms an eigenvalue problem which is symmetric, on account of Eq. (5) and it is left invariant by the transformation

13 $$ w \to - w,\,z \to - z,\,R_{T} \to - R_{T} ,R \to - R,\,\alpha \to - \alpha ,\,\beta \to - \beta . $$

## Computation of the eigenvalue

The resulting eigenvalue problem can be solved with high numerical accuracy by employing the Chebyshev collocation method. This mathematical technique is well documented in the literature (Canuto et al.^25^). In the collocation method, the *k*th order Chebyshev polynomial is

14 $$ \xi_{k} (z) = \cos (k\cos^{ - 1} z), $$

and the Chebyshev collocation points are

15 $$ z_{j} = \cos (\pi j/N),\quad j = 0,1,2, \ldots ,N, $$

where $j = 0$ and $j = N$ correspond to the upper and lower wall boundaries, respectively. The field variable *w* is approximated in terms of Chebyshev polynomials as

16 $$ w(z) = \sum\limits_{j = 0}^{N} {w_{j} } \,\xi_{j} (z), $$

where $w_{j}$ is a constant. Using (16), the stability Eqs. (11) and (12) are discretized in terms of Chebyshev polynomials to get

17 $$ \begin{aligned} & \left( {\sum\limits_{k = 0}^{N} {E_{jk} w_{k} + \left( {\alpha^{2} + \beta^{2} } \right)^{2} w_{j} - 2\left( {\alpha^{2} + \beta^{2} } \right)\sum\limits_{k = 0}^{N} {B_{jk} w_{k} } } } \right) - R_{T} \left( {\sum\limits_{k = 0}^{N} {C_{jk} w_{k} - \left( {\alpha^{2} + \beta^{2} } \right)\sum\limits_{k = 0}^{N} {A_{jk} w_{k} } } } \right) \\ & \quad = i\alpha R\left[ {\left( {U_{B} - c} \right)\left( {\sum\limits_{k = 0}^{N} {B_{jk} w_{k} - \left( {\alpha^{2} + \beta^{2} } \right)w_{j} } } \right) - D^{2} U_{B} w_{j} } \right],\quad j = 1(1)N - 1, \\ \end{aligned} $$

18 $$ w_{0} = w_{N} = 0, $$

19 $$ \sum\limits_{k = 0}^{N} {A_{jk} w_{k} } = 0,\,\,\,\,j = 0\, \& \,N, $$

where

$$ \begin{aligned} A_{00} & = \frac{{\left( {2N^{2} + 1} \right)}}{6} = - A_{NN} , \\ A_{jk} & = \frac{{c_{j} ( - 1)^{k + j} }}{{c_{k} (z_{j} - z_{k} )}};\quad j \ne k, \\ A_{jk} & = \frac{{z_{j} }}{{2(1 - z_{j}^{2} )}};\quad 1 \le j = k \le N - 1, \\ B_{jk} & = A_{jm} \cdot A_{mk} ,\quad C_{jk} = A_{jm} \cdot B_{mk} ,\quad E_{jk} = B_{jm} \cdot B_{mk} , \\ \end{aligned} $$

with

$$ c_{j} = \left\{ {\begin{array}{*{20}l} 2, \hfill & {j = 0,N} \hfill \\ 1, \hfill & {1 \le j \le N - 1} \hfill \\ \end{array} } \right. $$

The discretized equations lead to a generalized eigenvalue problem of the form

20 $$ {\mathbb{A}}X = c{\mathbb{B}}X, $$

where ${\mathbb{A}}$ and ${\mathbb{B}}$ are the square complex matrices of order $(N + 1)$ and *c* and *X* are the complex eigenvalue and the corresponding eigenvector, respectively. The above generalized eigenvalue problem usually describes the linear stability boundary of the basic flow, and the eigenvalues of the eigenvalue problem are calculated by using QZ algorithm (Moler and Stewart^26^). A suitable environment for the implementation of these steps is carried out using Mathematica 11.3 software (for a detailed procedure, see Shankar et al.^27–29^).

## Numerical results and discussion

The linear stability of plane Couette flow (PCF) in a horizontal layer with a uniform vertical throughflow is investigated numerically using the Chebyshev collocation method. The important non-dimensional parameters which govern the flow are the Reynolds number $R$ and the throughflow dependent Reynolds number $R_{T}$. The results are presented in terms of critical values of *R* computed with respect to the wave number $\alpha$ for various values of $R_{T}$. If water is the working fluid then its kinematic viscosity is $\nu = 0.801 \times 10^{ - 6} {{{\text{m}}^{{2}} } \mathord{\left/ {\vphantom {{{\text{m}}^{{2}} } {\text{s}}}} \right. \kern-\nulldelimiterspace} {\text{s}}}$, and if $W_{0}$ is in the range of 0.00001 to 0.002 m/s and $h = 10^{ - 2} \,{\text{m}}$ then the throughflow dependent Reynolds number $R_{T}$ lies in the range of 0.1 to 25.

### Validation of the code

The accuracy and the validity of our numerical code have been verified in two different ways. Firstly, the convergence was tested based on the response of number of collocation points (*N*) on the critical Reynolds number $R_{c}$, the corresponding critical wave number $\alpha_{c}$ and the critical wave speed $c_{c}$ for different values of throughflow Reynolds number $R_{T}$. The computed values are tabulated in Table 1 and note that the numerical accuracy improves with increasing *N* in Eq. (16). It can also be pointed out from the table that the numerical scheme needs a good number of collocation points to achieve the convergence with increasing $R_{T}$. Secondly, considering the formalism of Fransson and Alfredsson^21^ computations were carried out to validate the present numerical code and the results so obtained are compared in Table 2. It is seen that the results computed from the present code are in excellent agreement with the published ones.

**Table 1:** Convergence process of the Chebyshev collocation method for different values of $R_{T}$.

$N$	$R_{T} = 4$			$R_{T} = 5$			$R_{T} = 10$			$R_{T} = 15$			$R_{T} = 20$		
	$R_{c}$	$\alpha_{c}$	$c_{c}$	$R_{c}$	$\alpha_{c}$	$c_{c}$	$R_{c}$	$\alpha_{c}$	$c_{c}$	$R_{c}$	$\alpha_{c}$	$c_{c}$	$R_{c}$	$\alpha_{c}$	$c_{c}$
20	12,257.80	1.3942	0.4928	12,972.83	11.2334	0.6055	6435.12	11.9572	0.3562	4971.54	12.1271	0.1945	4856.29	11.8094	0.0871
30	76,776.23	1.7795	0.6970	48,599.68	2.2732	0.6417	31,756.79	2.9031	0.4782	21,138.88	25.9007	0.5085	15,902.50	26.5750	0.4011
40	154,576.83	0.6904	0.7119	96,559.55	0.9650	0.6723	69,476.20	4.5187	0.6096	45,447.30	5.6670	0.5021	51,368.57	44.6299	0.6040
50	194,297.45	0.4299	0.7052	144,531.45	0.7191	0.6921	228,717.15	1.9132	0.6908	115,417.98	6.8328	0.6199	77,986.35	8.3851	0.5422
60	194,190.06	0.4309	0.7053	167,175.00	0.6825	0.7008	243,757.38	1.5990	0.6919	274,981.47	2.9820	0.6745	189,689.03	9.1735	0.6399
70	194,187.32	0.4309	0.7053	167,237.93	0.6822	0.7008	286,849.61	1.5101	0.7034	510,929.92	2.3163	0.7163	490,738.92	8.7316	0.7150
80	194,187.07	0.4309	0.7053	167,238.84	0.6821	0.7008	272,963.22	1.5532	0.7000	379,068.91	2.4667	0.6954	605,815.20	3.4051	0.7095
90	194,187.07	0.4309	0.7053	167,238.56	0.6821	0.7008	273,592.16	1.5501	0.7002	415,956.46	2.2838	0.7015	481,042.36	3.2538	0.6915
100				167,238.56	0.6821	0.7008	273,587.66	1.5503	0.7002	408,315.60	2.3302	0.7003	565,848.57	3.1082	0.7032
110							273,584.48	1.5503	0.7002	407,928.88	2.3323	0.7002	548,169.80	3.1006	0.7008
120							273,584.48	1.5503	0.7002	407,953.59	2.3319	0.7002	542,168.78	3.1144	0.7000
130										407,949.88	2.3319	0.7002	544,305.41	3.1071	0.7003
140										407,949.84	2.3319	0.7002	543,796.90	3.1093	0.7002
150										407,949.84	2.3319	0.7002	543,790.15	3.1093	0.7002
160													543,793.40	3.1093	0.7002
170													543,792.74	3.1093	0.7002
180													543,792.74	3.1093	0.7002

**Table 2:** Comparison of $(R_{c} ,\alpha_{c} ,c_{c} )$ between the present study and Fransson and Alfredsson^21^ for different values of $R_{T}$.

$R_{T}$	Fransson and Alfredsson^21^			Present study		
	$R_{c}$	$\alpha_{c}$	$c_{c}$	$R_{c}$	$\alpha_{c}$	$c_{c}$
0	5772.22	1.02039	0.263982	5772.22	1.0205	0.2640
0.2	5967.01	1.01189	0.261378	5967.01	1.0119	0.2614
0.4	6607.4	0.99025	0.25399	6607.42	0.9902	0.2540
0.6	7902.5	0.95361	0.24148	7902.53	0.9537	0.2415

### Growth rate

**Figure 3:**
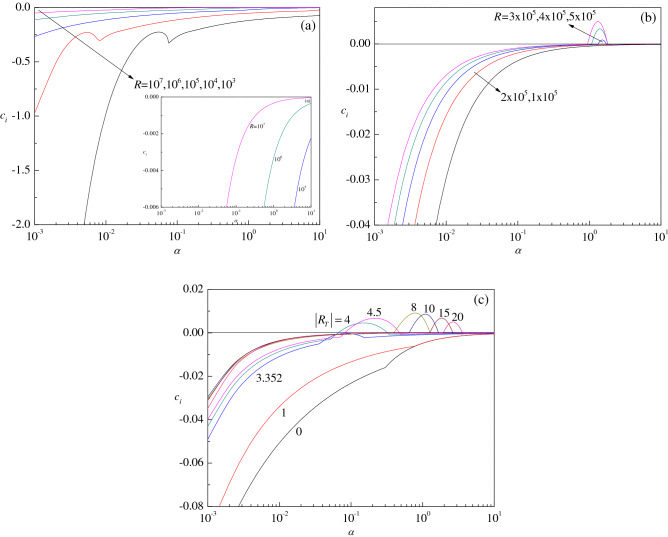
Plots of the growth rate $c_{i}$ versus wave number $\alpha$ for different values of (a) $R$ with $R_{T} = 0$ (absence of throughflow), (b) $R$ with $R_{T} = 10$ (presence of throughflow), and (c) $\left| {R_{T} } \right|$ with $R = 10^{6}$.

The sign of $c_{i}$ yields significant information about the stability characteristics of the fluid flow. To accomplish this, the growth rate $c_{i}$ versus the wave number $\alpha$ for different values of $R$ and $\left| {R_{T} } \right|$ is illustrated in Fig. 3a–c. In the absence of throughflow (i.e. $R_{T} = 0$), it is seen that $c_{i}$ is negative for all values of the Reynolds number considered indicating that no linear perturbation mode grows with time so that the basic flow is always linearly stable (Fig. 3a) confirming the result documented in Drazin and Reid^3^. For $R_{T} = 10$, however, it is observed that the sign of $c_{i}$ changes from negative to positive signaling the flow switching over from stable to unstable depending on the value of $R$ and the same is evident from Fig. 3b. A closer inspection of this figure reveals that larger values of $R$ are necessary for $c_{i}$ to become positive. Figure 3c demonstrates the variation of $c_{i}$ for different values of $\left| {R_{T} } \right|$ confirming the symmetry defined by Eq. (13) as the direction of throughflow produces no change in the figure. Although the flow is stable at lower values of $\left| {R_{T} } \right|$ as well as for $R_{T} = 0$ (i.e. absence of throughflow), it becomes unstable with increasing $\left| {R_{T} } \right|$. The above results clearly indicate that there is a possibility of PCF becoming unstable depending on the magnitude of throughflow.

### Neutral stability curves and critical values

**Figure 4:**
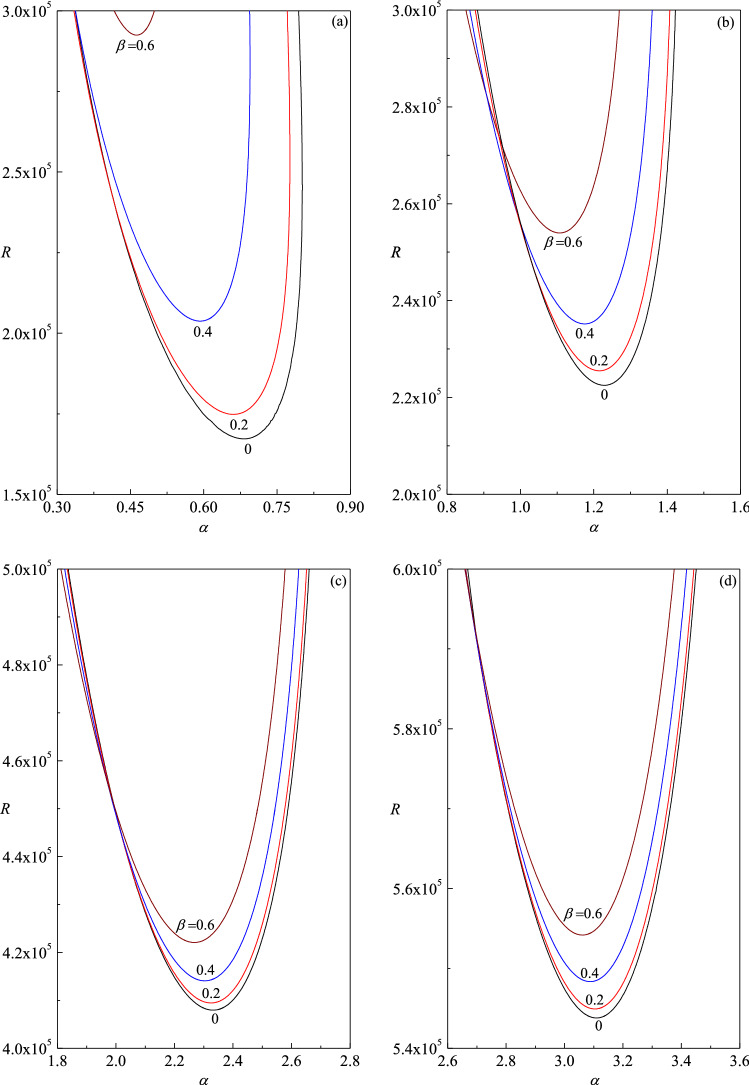
Neutral stability curves in the $(\alpha ,R)$-plane for different values of $\beta$ with (**a**) $R_{T} = 5$, (**b**) $R_{T} = 8$, (**c**) $R_{T} = 15$ and (**d**) $R_{T} = 20$.

The neutral stability curves in the $\left( {\alpha ,R} \right)$-plane are illustrated for different values of $\beta$ in Fig. 4a–d for $R_{T}$ = 5, 8, 15 and 20, respectively. The fundamental meaning is that the neutral stability condition ($c_{i} = 0$) is the bulk of a linear stability analysis as it provides the threshold between the linear stability and instability. For a given $\beta$, the unstable condition $\left( {c_{i} > 0} \right)$ lies in the region enclosed by the corresponding neutral stability curve. The region below and outside the curve is that defining the parametric conditions of linear stability $\left( {c_{i} < 0} \right)$. As a consequence, the absolute minimum of the neutral stability curve yields the so-called critical value of $\alpha ,\,R$ and $c$ which are denoted by $\alpha_{c} ,\,R_{c}$ and $c_{c}$, respectively. The physical meaning of the critical conditions is that no linear instability is possible for $R < R_{c}$. From the figures, it is intriguing to note that the two-dimensional disturbances ($\beta = 0$) are not the first to go unstable for all values of $\alpha$. A closer look at the figures, it is evident that three-dimensional disturbances ($\beta \ne 0$) become unstable earlier to that of $\beta = 0$ at lower range of values of $\alpha$ (Fig. 4b). Nonetheless, the observed trend reverses with increasing $\alpha$ and thereby the critical Reynolds number at which the instability triggers first turns out to be the least for only two-dimensional disturbances (i.e. validity of Squire’s theorem). The reason for this conflicting behavior may be attributed to the fact that we are not plotting the neutral curves against the overall wave number of the roll and probably one should consider the orientations of the roll as well to overcome this inconsistency.

**Figure 5:**
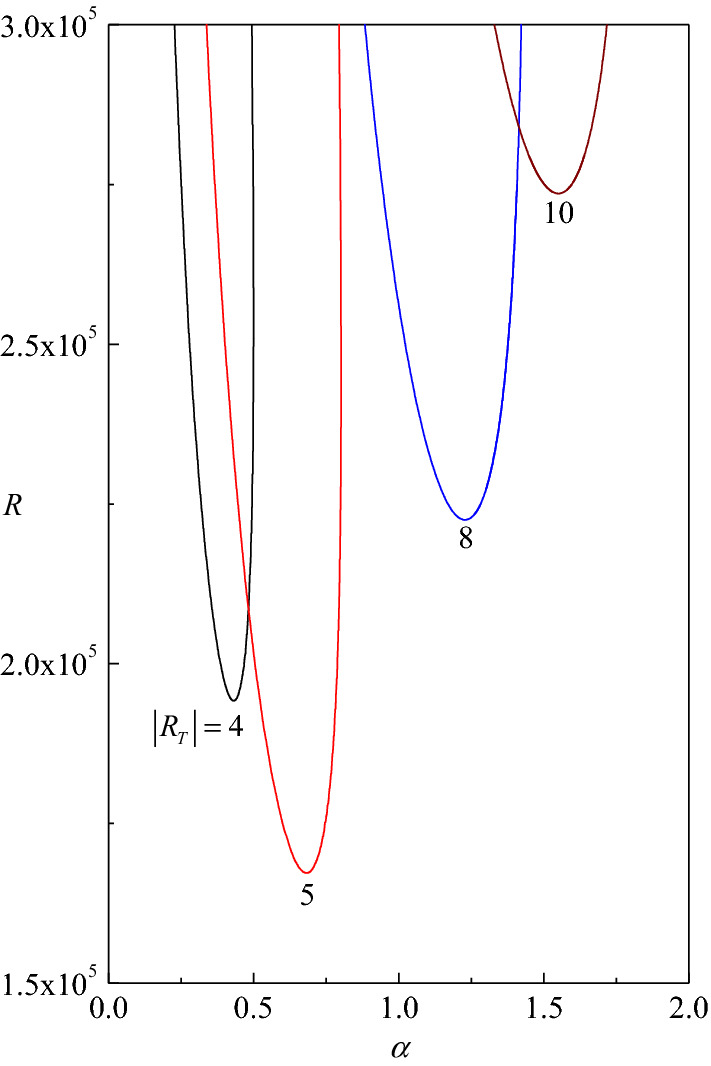
Neutral stability curves in the $(\alpha ,R)$-plane for different values of $R_{T}$ with $\beta = 0$.

Figure 5 shows the neutral stability curves for different values of $\left| {R_{T} } \right|$ with $\beta = 0$ and it is evident that the vertical throughflow ensures both stabilizing and destabilizing effect. This means that the neutral stability curve moves both downward and upward when $\left| {R_{T} } \right|$ increases so as the pushing of instability region. It is also observed that the normal modes activating the instability correspond to increasing wave numbers as $R_{T}$ increases, which physically means very small wavelengths. Moreover, the sign of critical wave speed $c_{c}$ remains to be positive (negative) for all positive (negative) values of $R_{T}$ indicating the perturbation modes are travelling.

### Stability of PCF

**Figure 6:**
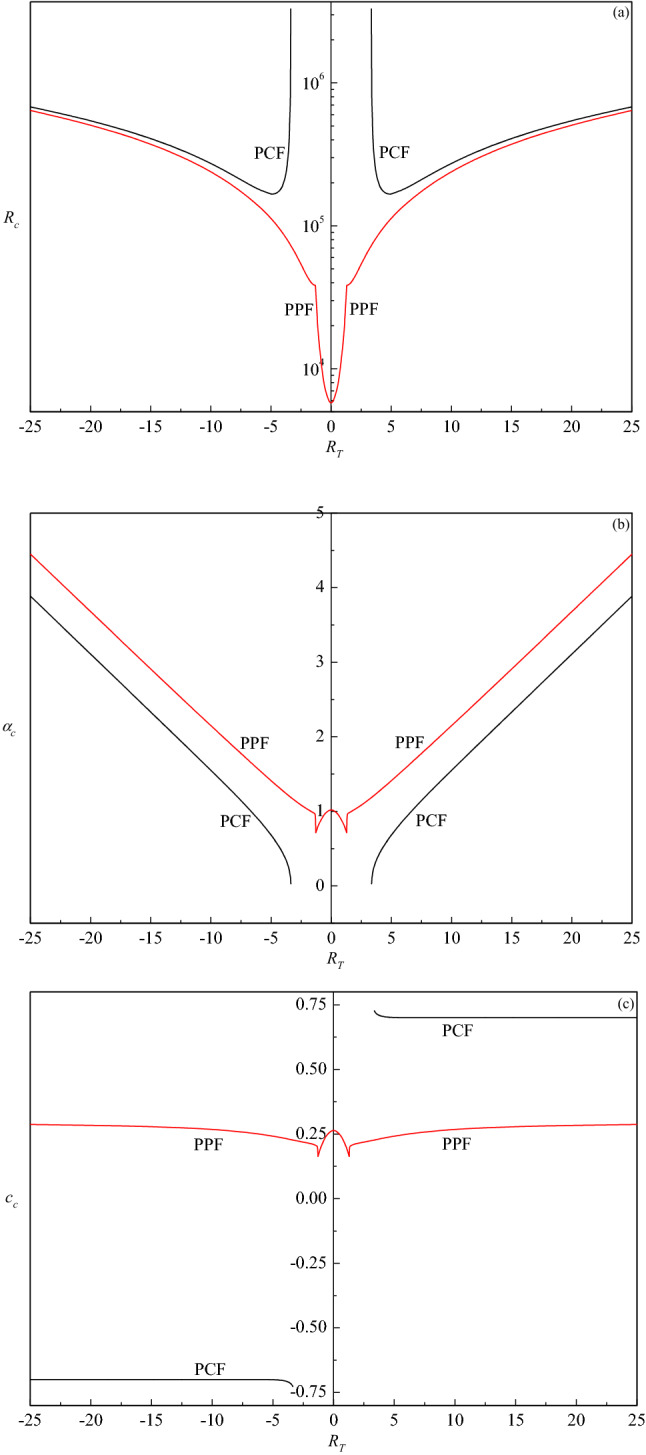
Plots of (**a**) $R_{c}$, (**b**) $\alpha_{c}$ and (**c**) $c_{c}$ as a function of $R_{T}$ for the plane Couette flow (PCF) and plane Poiseuille flow (PPF).

The critical triplets $\left( {R_{c} ,\alpha_{c} ,c_{c} } \right)$ for PCF are plotted in Fig. 6 as a function of $R_{T}$ and the results obtained for PPF are also shown for the sake of comparison. The direction of throughflow does not alter $R_{c}$ (Fig. 6a, Table 3) and hence it has no impact on the stability of fluid flow. From Fig. 6a, it is evident that there exists a threshold value of $\left| {R_{T} } \right|$= 3.353 prior to which the flow is always stable because the perturbations exhibit a negative growth rate as the throughflow effect is not strong enough to induce instability. Beyond this threshold value, however, a self-excited mode of disturbance exists for PCF; a result of contrast observed in the absence of throughflow (Drazin and Reid^3^). This may be due to the occurrence of Tollmien-Schlichting wave with increasing $\left| {R_{T} } \right|$. Moreover, the value of $R_{c}$ decreases rapidly and assumes a minimum value of 167,087.609 at $\left| {R_{T} } \right| = 4.899$. That is, there exists a range of $\left| {R_{T} } \right|$($3.353 \le \left| {R_{T} } \right| \le 4.899$) in which $R_{c}$ decreases (i.e. flow is destabilizing) but $R_{c}$ starts increasing (i.e. flow is stabilizing) again with further increase in $\left| {R_{T} } \right|$(> 4.899) and attains an asymptotic value 27,189.31 $\left| {R_{T} } \right|$ for $\left| {R_{T} } \right| > 25$. This may be due to the transport of momentum that opposes the stabilizing effect of viscous diffusion, and this is large when $\left| {R_{T} } \right|$ increases, and hence, higher values of $R_{c}$ are needed to initiate instability.

**Table 3:** Values of critical triplets $(R_{c} ,\alpha_{c} ,c_{c} )$ for different values of $R_{T}$.

$R_{T}$	$R_{c}$	$\alpha_{c}$	$c_{c}$	$N$	$R_{T}$	$R_{c}$	$\alpha_{c}$	$c_{c}$	$N$
4	194,187.07	0.4309	0.7053	90	− 4	194,187.07	0.4309	− 0.7053	90
5	167,238.56	0.6821	0.7008	100	− 5	167,238.56	0.6821	− 0.7008	100
10	273,584.48	1.5503	0.7002	120	− 10	273,584.48	1.5503	− 0.7002	120
15	407,949.84	2.3319	0.7002	150	− 15	407,949.84	2.3319	− 0.7002	150
20	543,792.74	3.1093	0.7002	180	− 20	543,792.74	3.1093	− 0.7002	180

The corresponding values of critical wave number $\alpha_{c}$ for PCF are presented in Fig. 6b. The critical wave number is independent of the direction of throughflow and an increasing function of $\left| {R_{T} } \right|$. In particular, $\alpha_{c}$ increases from $\left| {R_{T} } \right|$= 3.353 and shows a steady rise in their values, eventually attaining a limiting value $\alpha_{c} = 0.155469\left| {R_{T} } \right|$ for sufficiently large values of $\left| {R_{T} } \right|$. We have shown that the principal effect of the vertical throughflow is to modify the steady velocity distribution, which tends to exponential boundary-layer type with thickness of the order of ${\alpha \mathord{\left/ {\vphantom {\alpha {\left| {R_{T} } \right|}}} \right. \kern-\nulldelimiterspace} {\left| {R_{T} } \right|}}$ with increasing $\left| {R_{T} } \right|$. Therefore, we may expect that the critical wavelength will be of the same order of magnitude as the boundary-layer thickness. Thus the critical wave number will increase with increasing $\left| {R_{T} } \right|$ indicating the effect of throughflow is to reduce the size of convection cells.

The curves of critical wave speed $c_{c}$ start from the value of $\left| {R_{T} } \right|$ beyond which the flow becomes unstable (Fig. 6c). Initially, the curve of $c_{c}$ exhibits a decreasing trend and thereafter remains invariant with further increase in $\left| {R_{T} } \right|$. The sign of $c_{c}$ becomes negative (positive) when $\left| {R_{T} } \right|$ is negative (positive) but its modulus does not change. Thus the cells move up (down) the layer when the solutions correspond to $c_{c} < 0$ $\left( {c_{c} > 0} \right)$.

### Comparative study between PPF and PCF

In the case of PPF both the plates are at rest and an external pressure gradient drives the flow in the horizontal direction whereas in PCF both the plates are considered to be moving in the opposite directions with a uniform speed $U_{0}$, and the relative motion drives the shearing action in the fluid between the plates. As a result, the basic velocity for these two flows is found to be different due to asymmetric boundary conditions. However, the resulting stability equation is the same for both PPF and PCF even in the presence of throughflow. In the case of no throughflow ($R_{T}$ = 0), the basic state becomes unstable when the Reynolds number is larger than 5772.22 (Orszag^1^) for PPF whereas in the case of PCF, the basic flow is always stable (Drazin and Reid^3^). It is observed from Fig. 6a that the critical Reynolds number of PPF lies well below the critical Reynolds number of PCF for all values of $\left| {R_{T} } \right|$, indicating PCF has a more stabilizing effect than PPF. The vertical throughflow, irrespective of its direction, imparts only stabilizing effect on PPF, in general. In contrast, the throughflow indicts both destabilizing and stabilizing effect on PCF. The critical wave numbers for PPF are higher than PCF, indicating that the size of the cell width of PPF is smaller than that of PCF throughout the domain of $\left| {R_{T} } \right|$ (Fig. 6b). The critical wave speed values of PPF are lower than those of PCF for upward throughflow, while an opposite trend is observed for downward throughflow (Fig. 6c). Although the sign of $c_{c}$ remains positive for all values of $\left| {R_{T} } \right|$ for PPF, its sign changes for PCF and it depends on the direction of throughflow as well. Figure 6b,c also show that there is an abrupt variation in their values in the case of PPF and this may be due to the occurrence of bi-modal structure within a limited range of $\left| {R_{T} } \right|$ in the neutral stability curve. In the beginning, the left minimum (lower wave number) determines the critical mode; however, a slight increase in $\left| {R_{T} } \right|$ from 1.29 to 1.3 makes the right minimum (higher wave number) to be lower than the left one to dominate the instability. The switching over of instability mode is the cause for this sudden change in these critical values. While in the case of PCF uni-modal structure is observed and hence there is no change in the mode of instability.

## Conclusions

The change in the hydrodynamic stability of plane Couette flow (PCF) due to the presence of a uniform vertical throughflow has been investigated. A modal stability analysis of small-amplitude disturbances has been performed and the resulting stability eigenvalue problem is solved numerically using the Chebyshev collocation method. Some interesting and unexpected results are unveiled on the stability characteristics of the fluid flow. The numerical approach has shown that that three-dimensional disturbances are always more stable than two-dimensional ones. Suitable symmetries in the stability eigenvalue problem is found allowing one to gather information on the regime $\left| {R_{T} } \right| < 0$ from the results of $\left| {R_{T} } \right| > 0$. It is found that PCF is always stable in the absence of throughflow and also for values of $\left| {R_{T} } \right| \le 3.352$ and beyond this value it becomes unstable as the growth rate turns out to be positive from negative. In the parameter space $3.353 \le \left| {R_{T} } \right| \le 4.899$, the flow gets destabilized manifesting itself as a minimum in the $(R_{T} ,R_{c} )$-plane and stabilized reversely for values of $\left| {R_{T} } \right| > 4.899$ and attains an asymptotic value 27,189.31 $\left| {R_{T} } \right|$ for sufficiently large values of $\left| {R_{T} } \right|$. Besides, the instability is found to set in always via travelling-wave mode and PCF has a more stabilizing effect than PPF in the presence of vertical throughflow. The size of convection cells decrease with increasing $\left| {R_{T} } \right|$ and the cells may move up ($c_{c} < 0$) or down $(c_{c} > 0)$ the layer depending on the direction of throughflow.

## Data Availability

The data that supports the findings of this study are available within the article.
